# Vitamin B_12_ Supplementation and NT-proBNP Levels in COPD Patients: A Secondary Analysis of a Randomized and Controlled Study in Rehabilitation

**DOI:** 10.3389/fnins.2020.00740

**Published:** 2020-07-14

**Authors:** Fernanda Viana Paulin, Leandro Steinhorst Goelzer, Paulo de Tarso Müller

**Affiliations:** Laboratory of Respiratory Pathophysiology, Respiratory Division, Department of Medicine, Federal University of Mato Grosso do Sul, Campo Grande, Brazil

**Keywords:** COPD, exercise training, hyperhomocysteinemia, natriuretic peptides, vitamin B_12_

## Abstract

**Purpose:**

There is evidence of complex interaction between vitamin B_12_ (vB_12_) level, hyperhomocysteinemia (HyCy), and natriuretic peptide secretion. Exercise training could also modulate such interaction. In this secondary analysis of a Randomized Clinical Trial performed in a chronic obstructive pulmonary disease (COPD) rehabilitation setting, our primary objective was to investigate the interaction between vB_12_ supplementation, exercise training, and changes in NT-proBNP levels after 8 weeks of intervention. Secondary objectives were to explore the correlations between acute changes in NT-proBNP levels with (i) acute exercise and (ii) oxygen uptake (*V*’O_2_) kinetics during rest-to-exercise transition.

**Methods:**

Thirty-two subjects with COPD were randomized into four groups: Rehabilitation+vB_12_ (*n* = 8), Rehabilitation (*n* = 8), vB_12_ (*n* = 8), or Maltodextrin(*n* = 8). They were evaluated at baseline and after 8 weeks, during resting and immediately after maximal exercise constant work-rate tests (CWTs, *T*lim), for NT-proBNP plasmatic levels.

**Results:**

After interaction analysis, the supplementation with vB_12_ significantly changed the time course of NT-proBNP responses during treatment (*p* = 0.048). However, the final analysis could not support a significant change in NT-proBNP levels owing to high-intensity constant work-rate exercise (*p*-value > 0.05). There was a statistically significant correlation between *V*’O_2_ time constant and ΔNT-proBNP values (*T*lim – rest) at baseline (*p* = 0.049) and 2 months later (*p* = 0.015), considering all subjects (*n* = 32).

**Conclusion:**

We conclude that vB_12_ supplementation could modulate NT-proBNP secretion. Moreover, possibly, the slower the initial *V*’O_2_ adjustments toward a steady-state during rest-to-exercise transitions, the more severe the ventricular chamber volume/pressure stress recruitment, expressed through higher NT-proBNP secretion in subjects with larger *V*’O_2_ time constants, despite unchanged final acute exercise-induced neurohormone secretion.

## Introduction

In a recent Randomized Controlled Trial (RCT), we showed a slight but significant increase in maximal exercise tolerance (*T*lim) in patients with chronic obstructive pulmonary disease (COPD) supplemented with vitamin B_12_ during physical training, but without effects on oxygen uptake (*V*’O_2_) kinetics beyond training alone (Paulin et al., 2017). Individuals with COPD are at risk of vitamin B_12_ deficiency (Solomon, 2016) and hyperhomocysteinemia (HyCy) (Seemungal et al., 2007; Fimognari et al., 2009). Accordingly, HyCy is linked to numerous cardiovascular alteration (Ganguly and Alam, 2015) including histological changes in the heart (Piquereau et al., 2017), impaired global and segmental cardiac contractility (Kaya et al., 2014), or increased N-terminal-pro-B-type natriuretic peptide (NT-proBNP) secretion (Herrmann et al., 2007; Guéant Rodriguez et al., 2013). This prepropeptide is synthesized and stored as a high molecular weight mass propeptide from both the atria and ventricles, and released mainly under pressure/volume overload of the cardiac chambers, after cleavage of the active form of BNP, inducing natriuresis and vasodilatation (Calzetta et al., 2016). However, NT-proBNP has a longer plasma half-life and attains larger concentrations, besides being described as a significant marker of prognosis in heart failure (HF) (Cipriano et al., 2014). Moreover, vitamin B_12_ or folic acid supplementation reduced NT-proBNP levels after 2 months of supplementation in subjects with NT-proBNP > 40 pg/mL (Herrmann et al., 2007), mitigated HyCy-induced cardiac dysfunction (Jeremic et al., 2018), and proved to be protective for mitochondrial function and cardiac contractile properties in a murine model, with lessening of upregulated atrial brain natriuretic peptide (ANP) (Piquereau et al., 2017). In contrast, an experimental study was negative for cardiac morphological alterations during HyCy induction (Taban-Shomal et al., 2009).

It is recognized that physical training causes a reduction in cardiac natriuretic peptides in HF (Cipriano et al., 2014) as well as which, a beneficial interaction between physical training and vitamin B_12_ or folate supplementation in reducing HyCy has been suggested (König et al., 2003; Tyagi and Joshua, 2014). Of note, there is evidence of increased secretion of BNP associated with increased pulmonary vascular resistance during acute exercise in COPD (Fujii et al., 1999); a mechanism which demonstrates potential for attenuation through physical training. Thus, this secondary analysis aims primarily to explore the interaction between vitamin B_12_ supplementation, physical training, and resting/exercise levels for NT-proBNP in a stable population of COPD patients. In addition, we sought to analyze possible associations between acute changes in NT-proBNP levels during exercise with *T*lim, delivered power (watts, w), and oxygen uptake kinetics (*V*’O_2_ time constant) on an ergometer, in order to explore the determinants of these possible changes. The central hypothesis was that there would be attenuation of neurohormone alterations with supplementation alone or combined with physical training.

## Materials and Methods

As this is a secondary study of an already published RCT, the entire methodology has been previously described in detail (Paulin et al., 2017). Similarly, ethical considerations and consent details are published and recorded in the Brasilian Clinical Trials Registry (ReBEC number RBR-55f97c/2014). Additional unpublished methods will be considered in this exploratory study.

### Participants and Study Design

In the final analysis, 32 stable COPD patients were consecutively randomized to four groups: (1) 8-weeks physical rehabilitation (REHA) group, (2) 8-weeks physical rehabilitation group with daily vitamin B_12_ supplementation of 500 mg (REHA+B_12_), (3) supplementation group as stand-alone with daily vitamin B_12_ supplementation of 500 mg (B_12_), and (4) placebo group (maltodextrin 500 mg) (P). All groups continued with their usual optimized pharmacological treatment for COPD. Among the subjects who completed the study, 28/32 were already being followed up at the specialized COPD clinic and had an echocardiogram performed within the previous 6 months. Mild mitral or aortic reflux and ventricular hypertrophy were accepted in the inclusion criteria. Patient history and further detailed physical examinations did not show signs of associated heart disease in the remaining subjects recruited without echocardiography.

### Standard Doppler Echocardiography

All transthoracic echocardiography followed standard guidelines (Lang et al., 2005). Measurements of the cardiac cavities, interventricular septum, and left ventricular posterior wall thickness were collected by M-mode and two-dimensional analysis. Left atrial volume used the biplane Simpson method and was indexed by body surface. The ejection fraction was measured by the Teichholz method. The tricuspid reflux velocity was obtained by continuous Doppler in the right ventricle inlet, and the sPAP value was calculated by adding 10 mmHg of pressure in the right atrium.

### Cardiopulmonary Exercise Testing (CPET)

**FIGURE 1:**
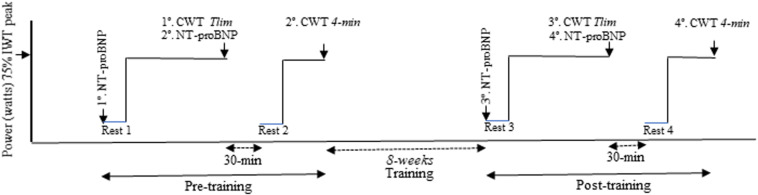
Exercise protocol during constant work test (CWT) before and after 8 weeks.

All subjects were invited to perform an incremental cardiopulmonary exercise testing (CPET) and two equal constant work-rate tests (CWTs, 75% of the maximum incremental CPET load) to calculate time constants (*tau*, τ) for *V*’O_2_ during the rest-to-exercise transition. Detailed CWT methods and oxygen uptake kinetics analysis were previously published (Paulin et al., 2017; Müller et al., 2019). Blood samples were collected at rest and *T*lim during the first CWT, before and after 8 weeks, for NT-proBNP plasmatic level analysis (Figure 1).

### NT-proBNP Analysis

After a suitable rest period, before the CWT, a polyurethane 22G catheter (Injex-Cath, Ourinhos, Brazil) was inserted on the back of the patient’s right hand for venous blood sample collections at rest and during exercise. The material was collected in tubes containing a mixture of plasma-lithium, as recommended by the manufacturer, and incubated at -20°C for 30 min. The electrochemiluminescence sandwich immunoassay by COBAS e602^®^ system (Roche Diagnostics, Germany) measurement method was used, with a measurement range of 5–35,000 pg/mL and predicted coefficient of variation < 3.1%.

### Data Analysis and Statistics

Data are expressed as mean ± standard deviation (SD) or median (IQR), mirroring, respectively, Gaussian or non-Gaussian distribution after the Shapiro–Wilk test. As τ (time constant, *tau*)—representing the time to attain 63% of the steady-state *V*’O_2_ during a CWT—demonstrates marked skewness, we performed a reciprocal transformation and used *k* (*k* = 1/τ) instead of τ. In exponential growth, *k* is equal to the reciprocal of the time constant *tau*, i.e., *k* = 1/τ and *represents the rate of increase in V*’O_2_ *toward a steady-state plateau*. *Hence, the faster the rate of adjustment (k) of V’O_2_ toward the steady-state during rest-to-exercise constant work-rate exercise transitions, the lower the time constant tau*. Thus, we chose to perform Pearson product-moment coefficients for correlations using *k*, as *k* generates a more robust Gaussian distribution (Bland and Altman, 1996) and *would imply similarly to a plausible physiological significance, as we recently suggested* (Müller et al., 2019). The slopes of linear regression for pre- and post-training moments were compared with Univariate ANCOVA. In the temporal analysis of the effects of training and supplementation, we performed three-way repeated measures (RM) ANOVA with two between-subjects factors (training and supplementation) and four within-subjects RM (NT-proBNP level at rest/*T*lim at baseline and rest/*T*lim after 2 months), taking into account the sphericity standard and the Greenhouse-Geisser correction. In addition, one-way ANOVA for groups was performed for comparison of baseline characteristics and one-way RM ANOVA for time-dependent within-group NT-proBNP level changes. The calculated study power > 0.8, with an alpha risk of 0.05 (two-tailed), was calculated from the placebo Group with their respective SD differences. Thus, we considered the average within-subjects SD difference of 1 pg/mL for NT-proBNP and, two between-subjects factors, B_12_ and exercise, with measured SD of the difference of 103 pg/mL and 100 s, respectively, in a three-way RM ANOVA design. We defined a *p*-value < 0.05 as statistically significant. The statistical program SPSS 20.0 was used for all statistical analysis (SPSS, IBM Corp, United States, 2011).

## Results

**FIGURE 2:**
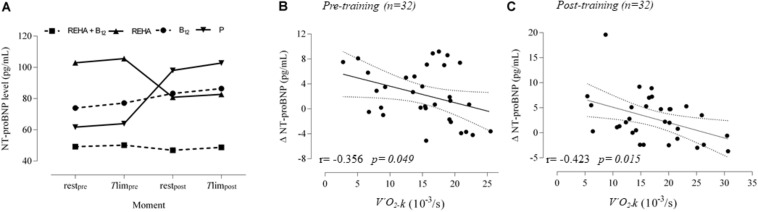
NT-proBNP levels at rest and *T*lim, before and after 2 months. The time course is depicted in **(A)** (interrupted lines were constructed only to show the time course of NT-proBNP level under vitamin B12 influence compared to control groups). Correlations for acute responses (*T*lim – rest, Δ) *vs* individual time constant for *V’*O_2_ (*k or 1/tau*) at baseline and 2 months later are represented in **(B)** and **(C)**. During the rest-to-exercise transition following constant work-rate exercise, there is an oxygen deficit that is inversely related to the rate of increase in*V’*O_2_ (*k*) toward the steady-state condition.

Selected baseline characteristics are described in Table 1 and detailed data have been previously published (Paulin et al., 2017). The groups were relatively balanced, with a significant difference only for *T*lim at baseline (*p* = 0.041, Table 1). Despite a similar baseline PaO_2_, the groups as a whole (*n* = 32) presented significantly reduced hemoglobin saturation through peripheral oximetry post-exercise (*p* < 0.0001). After interaction analysis, the supplementation with vitamin B_12_ significantly changed the time course of NT-proBNP level during treatment (*p* = 0.048, Table 2 and Figure 2A). In addition, the final analysis did not support a significant change in NT-proBNP levels owing to high-intensity constant work-rate exercise (three-way RM ANOVA analysis and one-way RM ANOVA analysis with *p*-value > 0.05 for both, Table 2). A statistically significant correlation was observed only between *V’*O_2_ time constant and ΔNT-proBNP level (*T*lim – rest) at baseline (*p* = 0.049, Figure 2B) and 2 months later (*p* = 0.015, Figure 2C), considering all subjects (*n* = 32), with an absence of significant correlations between ΔNT-proBNP level and *T*lim or delivered Power on the cycle ergometer (*p* > 0.05 for both). The pre- and post-training slopes representing the correlation between the *V’*O_2_ time constant and ΔNT-proBNP (Figures 2B,C) were not significantly different for the groups as a whole (*p* = 0.259).

**TABLE 1:** Selected baseline clinical and physiological characteristic of the four groups of COPD patients.

	REHA+B_12_	REHA+P	B_12_	P	*p*-value
**Subjects, n**	8	8	8	8	1.000
**Antropometry**					
Age (years)	56.55.0	65.26.0	63.45.2	58.110.3	0.156
Gender (M/F)	3/5	3/5	5/3	5/3	0.289
BMI (kg/m^2^)	24.52.9	25.16.0	24.54.0	28.35.5	0.332
*Lung function*					
FVC, % pred	65.216.0	72.210.0	66.013.0	73.013.1	0.534
FEV_1_, % pred	34.011.0	39.26.8	32.87.6	41.712.3	0.235
FEV_1_/FVC, %	40.710.4	43.59.2	41.211.4	44.96.8	0.695
PaO_2_, mmHg, rest	66.29.3	73.89.1	76.416.0	75.57.6	0.348
**CPET data**					
*V’*O_2peak_, % pred	50.516.1	65.117.7	68.818.2	64.718.2	0.191
W_peak_, % pred	43.612.4	46.813.1	34.712.0	43.115.2	0.324
*T*_lim_, s	410311	314230	259110^∗^	436143	**0.041**
τ, s	6537	8439	6922	6925	0.617
*k, 10^–3^/s* **Blood analysis**	17.77.1	13.16.2	14.34.5	15.74.4	0.443
Vitamin B12 (pre), pg/mL	385207	454309	451215	467114	0.877
Vitamin B12 (post), pg/mL	567227^†^	358177	544145	442204	0.148
ΔVitamin B12, pg/mL	182206	−71175	93262	−16103	0.060
Vitamin B12 < 300 pg/mL, *n*	3	4	3	1	0.340
Creatinine, mg%	0.80.2	0.90.2	0.80.1	0.80.1	0.314
Hematocrit, %	473	464	435	433	0.118
Hemoglobin, g %	151	151	142	141	0.102
**Echocardiography**					
LVM, g/m^2^	15622	13221	10817	12235	0.332
EF, %	703.8	662.1	694.8	654.2	0.126
LA, mm	361.4	374.5	301.2	312.4	0.201
LVDD, mm	462.1	491.9	452.1	442.9	0.133
PAP, mmHg	322.1	291.7	321.9	292.3	0.070

Abbreviations: BMI, body mass index; FVC, forced vital capacity; FEV_1_, forced expiratory volume in 1 s; DL_co_, carbon monoxide diffusion capacity; PaO_2_, partial pressure of oxygen in arterial blood; V’O_2peak__’_, peak oxygen consumption; T_lim_, maximal tolerance exercise time; τ, time constant (tau); TD, time delay; k, rate of V’O_2_ increase toward steady-state; LVM, left ventricular mass; EF, ejection fraction; LA, left atrium; LVDD, left ventricular diastolic diameter; PAP, pulmonary arterial pressure. *p < 0.05 B_12_ vs P; ^†^p < 0.05 B_12_ pre vs B_12_ post (Group REHA+B_12_). The Bold is only to highlight significant values, i.e, p-value < 0.05.

**TABLE 2:** NT-proBNP levels during acute exercise (rest and *T*lim) evaluated at baseline and after 2 months of intervention.

Group	Test time				One-way RM ANOVA^1^ *p*-value	Three-way RM ANOVA *p*-value
	Baseline		2 Months			
	Rest	*T*lim	Rest	*T*lim		
**REHA+B_12_**	41.1 (25.8–77.1)	42.7 (23.7–82.3)	50.1 (30.1–64.1)	51.0 (30.2–69.4)	0.958	0.075
**REHA+P**	95.0 (43.9–140.4)	100.3 (45.2–146.8)	49.5 (21.8–157.1)	50.5 (25.9–157.0)	0.204	0.917
**B_12_**	55.9 (41.1–121.8)	60.9 (43.7–126.8)	72.9 (40.8–134.7)	75.7 (47.6–133.2)	0.639	**0.048**
**P**	20.7 (18.8–111.6)	22.1 (20.1–118.2)	74.4 (21.9–175.1)	75.3 (25.2–177.6)	0.120	0.525
**Average (min-max)**	49.5 (27.9–123.3)	51.0 (24.2–130.4)	64.0 (27.8–99.4)	68.9 (29.7–105.0)	–	–
One-wayANOVA^2^ *p*-value	0.090	0.127	0.400	0.372		

*Abbreviations: REHA, rehabilitation; B_12_, vitamin B_12_; P, placebo; Tlim, time for maximal exercise tolerance. ^1^within-subjects one-way RM ANOVA; ^2^between-subjects one-way RM ANOVA. The Bold is only to highlight significant values, i.e, p-value < 0.05.*

## Discussion

This exploratory study suggests that B_12_ measurements and/or supplementation should be considered in future studies on cardiovascular morbidity in COPD subjects. This is in line with the overall pathophysiology of cardiovascular morbidity in COPD which has not yet been totally unraveled. In addition, we did not detect acute changes in plasma levels for NT-proBNP during high-intensity constant work-rate exercise. However, during the rest-to-exercise transition, patients with slower *V’*O_2_ adjustments toward a steady-state appeared to secrete higher NT-proBNP levels.

Normal levels of vitamin B_12_ do not rule out the possibility of cobalamin deficiency, and *low-normal* levels (200–300 pg/mL), when associated with risk factors for increased oxidative stress, demonstrate metabolic evidence of deficiency, with high levels of HyCy or methylmalonic acid (MMA), since this vitamin is inactivated by oxidation (Solomon, 2016). Our study population contained at least three subjects in each group with levels < 300 pg/mL of vitamin B_12_ and supplementation significantly increased their levels after 8 weeks for the REHA+B_12_ group and not significantly for the B_12_ group (Paulin et al., 2017).

Several previously cited animal and human studies have described a relationship between vitamin B_12_ or folic acid level, HyCy, and NT-proBNP levels. Although preliminary, our study points to an attenuating effect on NT-proBNP secretion with vitamin B12 supplementation, in agreement with previous studies (Herrmann et al., 2007; Guéant Rodriguez et al., 2013), despite weak evidence. In monitoring the evolution of HF, attenuated or stable levels of NT-proBNP secretion were associated with fewer total cardiovascular events (Franke et al., 2011). Although the mechanisms underpinning this small but significant effect are largely unexplored, downregulation of natriuretic peptide production by reducing homocysteine and MMA accumulation under certain conditions is possible (Piquereau et al., 2017).

Two important secondary findings were described. Although we did not find an acute change in NT-proBNP owing to acute exercise during high-intensity CWT, there was a small but significant relationship between an acute change in this neurohormone (Δ) and oxygen uptake kinetics. Our study differs from a previous study, which showed increased secretion of BNP after exercise in a population similar to ours during CWT (Fujii et al., 1999). These contradictory results occurred despite both presenting significant hemoglobin desaturation during exercise. Arterial hypoxemia is a known trigger for natriuretic peptide secretion (Fujii et al., 1999). Surprisingly, our study agrees with another study for NT-proBNP also under CWT in mild-to-moderate COPD (Wang et al., 2011). Differences may reflect other factors, such as the secretion-to-metabolization ratio of natriuretic peptides and differences in exercise protocol. Our data suggest that much of the secretion of natriuretic peptides during CWT occurs at the time of initial adjustment during rest-to-exercise transitions, where the sudden change in the cardiac chamber pressure/volume condition is additionally stressed by the direct effect of transmural pressure, due to the abrupt increase in intrathoracic respiratory pressure swings, following the kinetics of the increase in pulmonary arterial pressure during the first minute and posterior decay during exercise (Lonsdorfer-Wolf et al., 2004). In this sense, our study is in agreement with a previous study that showed a direct relationship between BNP secretion and oxygen deficit during constant work-rate exercise in HF (Brunner-La Rocca et al., 1999).

As a limitation of this study, we cite the small number of individuals, notwithstanding this being partially tempered by the strong study design. In addition, we did not measure homocysteine and MMA levels. Four subjects were not evaluated by echocardiography; however, clinical data, NT-proBNP levels, and CPET analysis were not compatible with major heart disease.

## Conclusion

We suggest that vitamin B_12_ supplementation could modulate NT-proBNP secretion, but the effects are small and further studies are needed. Moreover, we did not find an increase in neurohormone level caused by acute exercise *per se*; however, there was an association with slower *V’*O_2_ adjustment during the rest-to-exercise transition. The significance of this neurohormone dynamic warrants more detailed studies, considering different modalities of exercise training, e.g., high-intensity 1-min bouts, with unexplored cardiac neuro- hormone signaling and unknown cardiovascular consequences.

## Data Availability Statement

The datasets generated for this study are available on request to the corresponding author.

## Ethics Statement

The studies involving human participants were reviewed and approved by the Human Research Ethics Committee, affiliated to Brazilian Clinical Trial Registry and Federal University of Mato Grosso Do Sul. The patients/participants provided their written informed consent to participate in this study.

## Author Contributions

PM contributed to study design, literature search, data collection, analysis of data, and manuscript preparation and review. FP contributed to literature search, data collection, analysis of data, and manuscript preparation and review. LG contributed to study design, literature search, data collection, analysis of data, and manuscript review. All authors contributed to the article and approved the submitted version.

## Conflict of Interest

The authors declare that the research was conducted in the absence of any commercial or financial relationships that could be construed as a potential conflict of interest.
